# Herbal Extracts and Natural Products in Alleviating Non-alcoholic Fatty Liver Disease via Activating Autophagy

**DOI:** 10.3389/fphar.2018.01459

**Published:** 2018-12-11

**Authors:** Li Zhang, Zemin Yao, Guang Ji

**Affiliations:** ^1^Institute of Digestive Diseases, China-Canada Center of Research for Digestive Diseases, Longhua Hospital, Shanghai University of Traditional Chinese Medicine, Shanghai, China; ^2^Department of Biochemistry, Microbiology and Immunology, Ottawa Institute of Systems Biology, University of Ottawa, Ottawa, ON, Canada

**Keywords:** autophagy, Chinese herbal medicine extracts, non-alcoholic fatty liver, steatosis, inflammation, oxidative stress, apoptosis

## Abstract

Non-alcoholic fatty liver disease (NAFLD) is the most common form of chronic liver disease world-wide, and currently therapeutic options for NAFLD are limited. Herbal medicine (HM) may offer an attractive alternative for the treatment of NAFLD. Recent years have witnessed a growing interest in the autophagy-inducing agents, and autophagy activation has been recognized as an efficient strategy in managing NAFLD and related complications. Pharmacological studies have demonstrated certain potential of HM extracts and natural products in inducing autophagy, which might contribute to the efficacy of HM in preventing and treating NAFLD. This review aims to summarize current understanding of mechanisms of HM extracts and natural products in preventing and treating NAFLD. Specially, we focused on mechanisms by which autophagy can target the main pathogenesis events associated with NAFLD, including hepatic steatosis, inflammation, oxidative stress, and apoptosis. It is hoped that this brief review can provide a general understanding of HM extracts and natural products in treating NAFLD, and raise awareness of potential clinical application of HM in general.

## Introduction

Non-alcoholic fatty liver disease (NAFLD) is the most common form of chronic liver disease around the world, affecting one third of population in certain areas (Younossi et al., 2016). It is universally acknowledged that sedentary lifestyle, in conjunction with food abundance in industrialized countries are the main causes of NAFLD (Farrell et al., 2013). Clinically, NAFLD covers a broad spectrum of liver abnormalities, ranging from simple steatosis, non-alcoholic steatohepatitis (NASH), fibrosis, to cirrhosis (Cobbina and Akhlaghi, 2017). The hallmark of NAFLD is characterized by excessive accumulation of fat deposits in the liver, resulting from causes other than alcohol abuse. While simple steatosis is considered pathologically benign, NASH often indicates liver injury that may progress into severe pathology. Although NAFLD is a major component of metabolic syndrome and chronic liver diseases, a satisfactory explanation of any pathological aspects of NAFLD is unavailable at the moment.

Herbal medicine (HM), an alternative approach in the treatment of NAFLD has drawn growing attention among practitioners. In China, HM accounts for the majority of treatments in traditional Chinese medicine (TCM); plant elements and extracts are nature products that by far the most common elements used clinically. Although being used initially as empirical prescription for individuals, some of HM has been supported by clinical evidence in the treatment of NAFLD (Pan et al., 2013; Yu et al., 2015). The extracts of HM have also shown benefits in alleviating NAFLD (Xu et al., 2015); clinical trials have obtained evidence of natural product from HM, such as berberine (Yan et al., 2015), resveratrol (Chen et al., 2015), and curcumin (Panahi et al., 2017) in improving NAFLD parameters. Recently, studies have suggested that HM extracts could induce autophagy (Hu et al., 2016), which might offer an explanation of the efficacy of HM in NAFLD therapy.

Autophagy has been inferred in the pathogenesis of NAFLD and lipid dysregulation (Ueno and Komatsu, 2017). It is generally accepted that autophagy is activated during the early stage of NAFLD, in response to acute increase in lipid availability, thus attenuates lipid accumulation within the liver. However, hepatic autophagy is impaired upon sustained availability of lipids, such as long-lasting high fat dieting (Ueno and Komatsu, 2017). Autophagy is considered as one of the pathways in lipid breakdown (lipophagy), and is intimately associated with metabolism of lipid droplets (Kwanten et al., 2014). Autophagy can be cutely activated by a variety of means, such as caloric restriction, physical exercise, rapamycin, AMP-activated protein kinase (AMPK)-targeting agents, and hydrogen sulfide. Invariably, autophagy activation is associated with attenuation of NASH as well as improvement of various metabolic parameters (e.g., body weight, circulating glucose or triglyceride levels, and insulin sensitivity) (Lee et al., 2017). Moreover, autophagy activation with carbamazepine can reduce hepatic fibrosis in a model of α1-antitrypsin deficiency-associated liver disease (Puls et al., 2013). In contrast, inhibition of autophagy with a Beclin 1-interacting negative regulator results in accelerated lipid accumulation and pathogenesis of NAFLD (Tanaka et al., 2016).

The role of HM extracts in inducing autophagy and its implication in tumorigenesis has been investigated extensively (Wang et al., 2017; Zhang et al., 2018). Increasing attention has begun to focus on HM extracts and their effects on autophagy in NAFLD pathogenesis and related hepatic and metabolic complications. This review summarizes the current knowledge on the interrelationship between autophagy, autophagy inducing effect of HM extracts or natural products, and NAFLD.

## Autophagy and the Regulating Molecules

Autophagy is a highly conserved self-digestion process, bring dispensable or potentially dangerous cytoplasmic material, such as damaged organelles and misfolded or unfolded proteins, to lysosomes for degradation. To date, at least three autophagy processes have been described, namely microautophagy, chaperone-mediated autophagy (CMA) and macroautophagy. Macroautophagy is by far the most extensively characterized autophagy process, whereas the macroautophagy and CMA processes are lesser understood. Thus, the term autophagy is generally referred to the macroautophagy process. Autophagy is fundamental in the preservation of organismal fitness, and is central to adaptation to stress, usually alleviating damage of cells exposed to infections or else nutritional, metabolic physical or chemical (Kim and Klionsky, 2000).

In eukaryotic cells, autophagy is initiated by the formation of autophagosomes and autolysosome that leads to lysosome-mediated degradation. The process of autophagosome formation involves three major steps: initiation, nucleation, and elongation (Klionsky and Emr, 2000). Once the autophagosome enclosed, it can fuse with lysosome in the cytoplasm and assemble into autolysosome. More than 30 autophagy-related genes (ATGs) are involved in autophagy process (Amaravadi et al., 2016). The initiation of autophagosome is controlled by the ULK1-Atg13-FIP200 complex (Chan, 2009; Chan et al., 2009). The nucleation step requires the Beclin-1-class III phosphatidylinositol 3 Kinase (PI3K) complex that includes Beclin-1, Vps34 (class III PI3K), Vps15, Atg14L/Barkor, and Ambra-1 (Mizushima and Komatsu, 2011). Two conjugation systems are involved in the elongation of autophagosomes. The Atg12-Atg5-Atg16 complex and the cleavage of light chain 3 (LC3)/Atg8 cascade, leading to the soluble form LC3-I, and later forms the autophagic double-membrane associated LC3-II protein that allowing the closure of the autophagic vacuole (Ohsumi, 2001). The appearance of LC3-II and the autophagic adaptors p62 are commonly used to monitor autophagy influx. Closed autophagosomes fuse with lysosomes to generate autolysosomes for degradation. A large number of factors/actors regulate the autophagosome-lysosome fusion. In the autophagosome-lysosome fusion process, soluble *N*-ethylmaleimide-sensitive factor attachment protein, cytoskeleton proteins, and small GTPases are involved (Yu et al., 2017).

Mechanistic target of rapamycin (mTOR) complex 1 (mTORC1) exerts prominent autophagy-suppressing functions by catalyzing the inactivating phosphorylation of ATG13 and ULK1 (Noda and Inagaki, 2015). Such an inhibition can be relieved upon the inactivation of mTORC1 by AMPK, which is sensitive to cAMP accumulation (a consequence of ATP consumption) and also catalyzes the phosphorylation of ULK1 and Beclin 1 (Niso-Santano et al., 2015; Selleck et al., 2015).

## Autophagy Inducing Hm Extracts and Natural Products on Hepatic Steatosis

Hepatic lipid accumulation is the most notable feature of NAFLD. The liver is not a *de factor* organ for lipid storage. Under normal physiological conditions, the amount of fat that the liver contains is less than 5% of its weight. Thus, excessive lipid accumulation within the liver, known as ectopic lipid accumulation, is hallmark of hepatic steatosis, a typical characteristic of NAFLD. In the liver, triglyceride and cholesterol esters are the main constitutes of lipid droplet, and autophagy is closely associated with lipid droplets metabolism. LC3-positive structures are seen to co-localize with lipid droplet markers in liver tissue (Shibata et al., 2009) and in cell lines (Shibata et al., 2010; Sinha et al., 2012, 2014). Lipid droplets have also been shown to associated with lysosomes (Tuohetahuntila et al., 2017). Defective autophagy of lipid droplets in hepatocytes has recently been identified as a possible pathophysiological mechanism of NAFLD (Tuohetahuntila et al., 2017; Zhang et al., 2017). Thus, activating autophagy might be a promising strategy to attenuate hepatic lipid accumulation.

Some HM extracts and natural products are considered to be effective in attenuating lipid accumulation via, at least partly, activating autophagy. Ginsenoside Rb2, one of the major ginsenosides in *Panax ginseng*, is able to prevent hepatic lipid accumulation in *db/db* mice, HepG2 cells and primary mouse hepatocytes (Huang et al., 2017). Rb2 could partly reverse the repression of autophagic pathways involving AMPK or silent information regulator 1 (SIRT1). Inhibition of AMPK or SIRT1 pathway thus blocked the beneficial effects of Rb2 (Huang et al., 2017), suggesting that the effect of Rb2 on alleviating hepatic steatosis may be achieved through autophagy induction.

Resveratrol, a natural polyphenol, has been reported to improve complications associated with NAFLD pathology. Administrate resveratrol (200 mg/kg bodyweight) to rats under high fat diet conditions significantly prevented hepatic steatosis and hepatocyte ballooning after 18-week treatment, along with the up-regulation of SIRT1 and autophagy markers LC3-II, Beclin 1, and P62 (Ding et al., 2017). Similarly, another study has also shown beneficial effects of resveratrol on hepatic steatosis, and demonstrated that the effect is achieved partially through inducing autophagy and the cAMP-PRKA-AMPK-SIRT1 signaling pathway (Zhang et al., 2015a). In methionine-choline-deficient diet (MCD)-induced NASH animals, 4-week resveratrol intervention significantly decrease lipid accumulation in the liver, along with the increase LC3-II levels and decrease P62 expressions (Ji et al., 2015). When the autophagy inhibitor, chloroquine (CQ) is added, the beneficial effects of resveratrol on AML12 cells are then abolished (Ji et al., 2015), suggesting that the effects of resveratrol are likely associated with autophagy activation.

Akebia saponin D (ASD), extracts from *Akebia quinata*, has been implicated in treating NAFLD. Administration of ASD in *ob/ob* mice results in a significantly decrease in hepatic steatosis and an accompanied increase in autophagic flux (e.g., increased expression of LC3-II and decreased P62 accumulation). In oleic acid stressed Buffalo rat liver cells, ASD also prevented excessive lipid droplets formation and increased autophagic flux, however, CQ or siRNA mediated ATG7 knockdown could abolish the effect (Gong et al., 2016), suggesting that ASD is an activator of autophagy and a possible candidate for treating NAFLD.

Bergamot polyphenol fraction (BPF), one of the dietary polyphenols, could strongly reduce hepatic steatosis and counteract the pathogenic increase of serum triglycerides, blood glucose and obesity in experimental rats (Parafati et al., 2015). Importantly, BPF can stimulate autophagy as suggested by increased levels of LC3 and Beclin 1, and concomitant reduction of SQSTM1/p62, suggesting autophagy stimulation (Parafati et al., 2015).

Capsaicin, an extract of *Capsicum annuum* and a common dietary supplement, has been shown to exert beneficial effects on NAFLD (Gong et al., 2016). Treatment of capsaicin in HepG2 cells results in activation of the transient receptor potential vanilloid 1 (TRPV1), which is accompanied with significantly increased the expression of autophagy-related proteins, such as LC3 II, Beclin 1, Atg5, and Atg7 (Li et al., 2013).

## Autophagy Inducing Hm Extracts and Natural Products on Oxidative Stress

Oxidative stress describes an imbalance between the systemic manifestation of reactive oxygen species (ROS) and a biological system’s ability to readily detoxify the reactive intermediates or to repair the resulting damage (Braud et al., 2017). Although mechanisms underlying NAFLD pathogenesis remain undefined, the widespread theories, such as “two hit hypothesis” and “multiple parallel hits” all centralized oxidative stress as a driving factor for NAFLD progression (James and Day, 1998; Tilg and Moschen, 2010). Since lipid oxidative mainly occurs in mitochondria, the excess ROS generated from oxidative stress conceivably would cause mitochondrial damage and subsequently dysfunction. As the formation of autophagosome is usually initiated from the membrane of mitochondria or endoplasmic reticulum (ER), the dysfunction of mitochondria presumably contribute to inhibition of autophagy. Supporting this assumption is a recent observation that mediating oxidative stress can efficiently stimulate autophagy (Tang et al., 2017).

Licorice is a popular herbal plant widely used in the treatment of various diseases including liver diseases (Jung et al., 2016). Glycycoumarin (GCM) is a representative of coumarin compounds isolated from licorice. It is reported that GCM could block mitochondrial activation in palmitate stressed cells (HepG2, AML-12, and L02) and MCD diet induced NASH mice, in line with the improved metabolic disorders, and the effects are partially associated with reactivation of the impaired autophagy (Zhang E. et al., 2016).

*Lycium barbarum* is one of the commonly used HM; *L. barbarum* polysaccharides (LBP) are the main components of *L. barbarum*, accounting for 20–50% of the total extracts. Studies showed that LBP could significantly inhibit oxidative stress in various animal models and cell lines (Zhu et al., 2015; Varoni et al., 2017). In high fat diet-induced NASH rat model, 4 weeks LBP treatment showed ameliorative effects on metabolic and inflammatory parameters of NASH (Xiao et al., 2014). In addition, certain autophagic markers, such as Atg5 and LC3II, were significantly up-regulated while certain autophagic negative regulators, such as p-mTOR and p62, became down-regulated (Xiao et al., 2014).

## Autophagy Inducing Hm Extracts and Natural Products on Apoptosis

Accumulation of unfolded proteins in the ER triggers an adaptive response, known as the unfolded protein response (UPR), to restore ER homeostasis (Song et al., 2017). The UPR pathway is also required to maintain hepatic functions. However, prolonged UPR leads to ER stress and pro-apoptotic transcription factor activation (Schroder and Sutcliffe, 2010). Apoptosis is inferred in the pathogenesis of NAFLD, whereas handling apoptosis is considered to be beneficial in alleviating NAFLD and related complications.

Garlic-derived S-allylmercaptocysteine (SAMC) has been shown to ameliorate hepatic injury in a NAFLD rat model (Xiao et al., 2013a). Administration of SAMC during NAFLD development could protect the liver from chronic injury and reduced the number of apoptotic cells in these rats (Xiao et al., 2013b). SAMC treatment could also enhance the expression of key autophagic markers in the liver, with a concomitant decrease in the activities of LKB1/AMPK and PI3K/Akt pathways as well as a decrease in the activity of antiautophagic regulator mTOR (Xiao et al., 2013b).

However, some other herbal extracts activate autophagy and apoptosis simultaneously. Schisandrin B (Sch B) is an active dibenzocyclooctadiene isolated from *Schisandrae fructus*, with a wide array of pharmacological activities (Gao et al., 2016; Ran et al., 2018). Sch B has been shown to exhibit potent proapoptotic and proautophagic effects in AML-12 and RAW 264.7 cells (Zhang et al., 2015b). The inhibition of PI3K/protein kinase B (Akt)/mTOR signaling pathway is thought to associated with the proautophagic activities of Sch B (Zhang et al., 2015b).

## Autophagy Inducing Hm Extracts and Natural Products on Inflammation

Inflammation is the typical pathological feature of NASH. Inflammation is frequently triggered by various signals, including pro-inflammatory cytokines and chemokines, that are released from injured hepatocytes and activated Kupffer cells (Liu et al., 2016). Suppression of autophagy has been observed in Kupffer cells in the steatotic liver, and Kupffer cells with low autophagy activity are sensitized to endotoxin and subsequent inflammatory process (Fukada et al., 2012).

Dioscin is a saponin extracted and isolated from *Polygonatum Zanlanscianense* Pamp. Dioscin has been shown to markedly decrease serum ALT and AST levels in animals with liver injury, and rehabilitate inflammation via decreasing the expression levels of interleukin-1β (IL-1β), interleukin-6 (IL-6), and tumor necrosis factor alpha (TNF-α) (Zhang W. et al., 2016). In addition, dioscin has been shown to suppress collagen synthesis (Liu et al., 2015). Further studies have shown that Dioscin significantly decrease the expression of p-mTOR/mTOR level and sequentially activate autophagy (Xu et al., 2017).

Resveratrol has also been shown to decrease inflammatory infiltration in the liver of MCD-induced NASH animals, and decreased serum levels of ALT, AST, IL-1β, IL-6, and TNF-α in these animals which were associated with autophagy (Ji et al., 2015). In addition, resveratrol is reported to restore liver injury via activating autophagy and suppressing NF-κB activation in experimental animals (Li et al., 2014), suggesting autophagy activation could serve as an anti-inflammatory strategy and have the potential to prevent NAFLD progression.

## Conclusion and Perspectives

**FIGURE 1:**
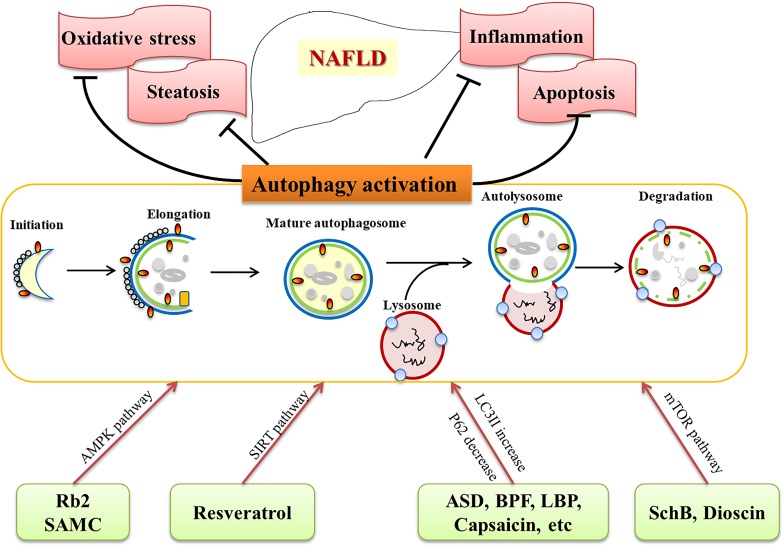
Herbal medicine (HM) extracts on NAFLD via autophagy activation. HM extracts and natural products activate autophagy through different pathways (AMPK, SIRT, mTOR, etc.), and target related pathological processes (steatosis, oxidative stress, apoptosis, inflammation) of NAFLD.

HM extracts and natural products are effective in treating NAFLD and related complications (Table 1), although the mechanisms are still under exploration. Recently studies provide valuable information on its role of HM extracts and natural products via activating autophagy. As discussed above, autophagy inducing agents are confirmed to be beneficial in NAFLD treatment in animal models and cell lines. The commonly used HM extracts and natural products, such as resveratrol, Rb2, dioscin, LBPs, GCM, are potential candidates for preventing and treating NAFLD/NASH, and biochemical and histological experiments have demonstrated their ability to activate autophagy (Figure 1). Numerous HM extracts and natural products are indicated in activating autophagy, however, the results should be interpreted with caution, since multiple targets are involved, and most of the data are obtained from studies with animals or cells, clinical evidence are urgently needed to evaluate the therapeutic effects and safety. Nevertheless, HM and natural products provide a promising choice, and activation of autophagy might be an underlying mechanism for the efficacy of the compounds.

**Table 1:** Herbal medicine and natural products that exert an effect on autophagy.

Herb name/active components	Targeted pathways	Reference
Ginsenoside Rb2	Activate AMPK and SIRT1 in *db/db* mice, HepG2 cells, and primary mouse hepatocytes	Huang et al., 2017
Resveratrol	Up-regulate SIRT1 and autophagy markers LC3-II, Beclin 1, and P62 in diet induced NAFLD animals	Ji et al., 2015; Zhang et al., 2015a; Ding et al., 2017
Akebia saponin D	Increase expression of LC3-II and decrease P62 accumulation in *ob/ob* mice and primary rat hepatocytes	Gong et al., 2016
Bergamot polyphenol fraction	Increase LC3 and Beclin 1 and reduce SQSTM1/p62 in diet induced NAFLD rats	Parafati et al., 2015
Capsaicin	Activate TRPV1 in HepG2 cells	Li et al., 2013
Glycycoumarin	Block mitochondrial activation in palmitate stressed cells and NASH animals	Zhang E. et al., 2016
*Lycium barbarum* polysaccharides	Activate Atg5 and LC3-II and down-regulate p-mTOR and p62 in NASH animals	Xiao et al., 2014
S-allylmercaptocysteine	Suppressed LKB1/AMPK and PI3K/Akt pathways in NAFLD rats	Xiao et al., 2013b
Schisandrin B	Inhibit PI3K/Akt/mTOR signaling pathway in AML-12 and RAW 264.7 cells	Zhang et al., 2015b
Dioscin	Decrease p-mTOR/mTOR in NASH animals	Xu et al., 2017

## Author Contributions

GJ proposed the topic and made the frame. LZ composed most of the context. GJ and ZY revised the manuscript.

## Conflict of Interest Statement

The authors declare that the research was conducted in the absence of any commercial or financial relationships that could be construed as a potential conflict of interest.
